# Notch-EGFR/HER2 Bidirectional Crosstalk in Breast Cancer

**DOI:** 10.3389/fonc.2014.00360

**Published:** 2014-12-12

**Authors:** Andrew T. Baker, Andrei Zlobin, Clodia Osipo

**Affiliations:** ^1^Integrative Cell Biology Program, Health Sciences Division, Cardinal Bernardin Cancer Center, Loyola University Chicago, Maywood, IL, USA; ^2^Health Sciences Division, Cardinal Bernardin Cancer Center, Loyola University Chicago, Maywood, IL, USA; ^3^Department of Pathology, Health Sciences Division, Cardinal Bernardin Cancer Center, Loyola University Chicago, Maywood, IL, USA

**Keywords:** Notch, EGFR, HER2, crosstalk, breast cancer

## Abstract

The Notch pathway is a well-established mediator of cell–cell communication that plays a critical role in stem cell survival, self-renewal, cell fate decisions, tumorigenesis, invasion, metastasis, and drug resistance in a variety of cancers. An interesting form of crosstalk exists between the Notch receptor and the Epidermal Growth Factor Receptor Tyrosine Kinase family, which consists of HER-1, -2, -3, and -4. Overexpression of HER and/or Notch occurs in several human cancers including brain, lung, breast, ovary, and skin making them potent oncogenes capable of advancing malignant disease. Continued assessment of interplay between these two critical signaling networks uncovers new insight into mechanisms used by HER-driven cancer cells to exploit Notch as a compensatory pathway. The compensatory Notch pathway maintains HER-induced downstream signals transmitted to pathways such as Mitogen Activated Protein Kinase and Phosphatidylinositol 3-Kinase (PI3K), thereby allowing cancer cells to survive molecular targeted therapies, undergo epithelial to mesenchymal transitioning, and increase cellular invasion. Uncovering the critical crosstalk between the HER and Notch pathways can lead to improved screening for the expression of these oncogenes enabling patients to optimize their personal treatment options and predict potential treatment resistance. This review will focus on the current state of crosstalk between the HER and Notch receptors and the effectiveness of current therapies targeting HER-driven cancers.

## Introduction

Current research has revealed many interesting features of cell signaling networks implicated in the development of breast cancer and continues to define methods by which cancer cells become able to exploit these networks to promote their growth, survival, and invasiveness. Communication between cell signaling pathways is critical to a cell’s response to an ever changing environment, and is harnessed by cancer cells to progress their disease state to obtain a growth advantage over healthy cells. Such pathways include the Epidermal Growth Factor Receptor (EGFR) Tyrosine Kinase (HER or HER) and Notch pathways, which have been found to communicate with one another to overcome treatment as well as promote Breast Cancer Stem Cell (BCSC) cell fate. This review presents current perspectives on research of HER–Notch crosstalk in breast cancer and culminates recent publications to give an up-to-date view of the intricate mechanisms that describe how communication between these two pathways is affecting the development of malignancies, drug resistance, recurrence, and metastatic progression.

## Breast Cancer at a Glance

1. Breast cancer is the second leading cause of cancer-related death among women worldwide. Women have a one in eight lifetime risk of being diagnosed with breast cancer in the US alone (1). Breast cancer is divided into four major subtypes based on receptor overexpression during tumorigenesis as well as a pre-invasive subtype known as ductal carcinoma *in situ* (DCIS). The subtypes of breast cancer include: luminal A [estrogen receptor (ER) +/ progesterone receptor (PR)+], luminal B (ER+, PR+, HER2/HER2±), and triple negative/basal-like (ER−, PR−, HER2−) (TNBC/BLBC) (2, 3). The luminal A, B, and HER2+ breast cancer subtypes use estrogen/progesterone and HER2 receptor overexpression and activation, respectively, to drive tumor growth while the TNBC subtype lacks comparable overexpression of these receptors (Table 1).
2. The luminal A and B breast cancer subtypes comprise 60–70% of all breast cancers and are derived from the luminal epithelium of breast ducts with cancer cells overexpressing the ER and/or PR. First line therapy for the Luminal A and B breast cancer subtypes are anti-hormonal therapies that target estrogen-mediated activation of the ERα isoform. Aromatase inhibitors (AI), tamoxifen, and fulvestrant are examples of anti-estrogen therapies, with the luminal A subtype being more sensitive to such inhibitors than the highly proliferative and inherently more resistant luminal B breast cancer subtype. The HER2/HER2+ breast cancer subtype has an amplification of the *HER2* gene. The *HER2* gene is a proto-oncogene and if mutated or overexpressed is a bonafide oncogene. *HER2* amplification results in overexpression of the HER2 receptor on the surface of breast cancer cell. The HER2+ subtype occurs in 20% of breast cancers and is sensitive to HER2-based-targeted therapies such as the humanized, monoclonal antibody trastuzumab, or the small molecule tyrosine kinase inhibitor, lapatinib. Mechanisms of action of these anti-HER2 therapies will be expounded later in this review. Among others, the TNBC/BLBC subtype does not express therapeutically targetable ER, PR, or HER2 receptors making this aggressive subtype difficult to treat. Since TNBC lacks expression of targetable receptors, treatment options for TNBC tumors are limited to cytotoxic chemotherapy such as tubulin-destabilizing taxanes (docetaxel, paclitaxel), DNA damaging alkylating/methylating agents (Cyclophosphamide, Chlorambucil, Temozolomide), or DNA untangling (topoisomerase II) stabilizers (doxorubicin, camptothecin) aimed at killing highly proliferative TNBC cells.
3. Early stage Ductal Carcinoma *In situ* (DCIS) is a non-invasive form of breast cancer in which luminal cells that line mammary gland ducts are morphologically and physiologically changed to resemble cancer cells, to some extent. If left untreated, DCIS can proceed to a metastatic disease in three steps. First, a population of cells in the lesion begins to fill in the hollow luminal space of the mammary duct. Second, these tumorigenic cells can invade the surrounding breast tissue to become Invasive ductal carcinoma. Third, the lesion becomes metastatic as the cancer cells begin to access blood or lymph circulation to invade distal parts of the body to form new tumors. If undiagnosed or left untreated, DCIS has a two in three chance of progressing to malignant disease (13). Molecular markers are similar between DCIS and invasive breast cancer and these include ER+ and HER2+. The expression of such markers can aid in determining a proper treatment regimen for DCIS (5). Treatments can range from molecular targeted therapies (tamoxifen, trastuzumab), radiation, surgery (lumpectomy or mastectomy), to a combination of surgery, radiation, and molecular targeted therapy with the end goal of breast conservation and reduced risk of subsequent invasive or *in situ* tumor recurrence (14, 15).
4. There are many different mutations that can cause breast cancer and this heterogeneity makes it a difficult disease to treat and at times, diagnose. Dramatic improvements have been made to treat and diagnose breast cancer with the best chance for survival continuing to be early detection. Persistent research of the inter-connected signaling pathways that cancer cells exploit for continued survival and proliferation has led to many interesting findings that may help improve breast cancer treatment. This review delves into recent research exploring the role of the Notch and HER/HER pathways in breast cancer and how these potentially complementary pathways are able to communicate with one another to promote breast cancer and/or tumor growth.

**Table 1:** **Breast cancer subtypes and therapies**.

Subtype	Molecular markers	First line therapy	Second line therapy	Approximate occurrence (%)	Reviewed in reference
DCIS	ER±HER2±	Surgery, radiotherapy, chemotherapy, none	Adjuvant to chemo/radiotherapy such as tamoxifen (ER+) or trastuzumab (HER2+), none	20	Narod (4), Leonard (5), Wiechmann (6)
Luminal A	ER+/PR+	Tamoxifen (pre or post menopausal)	Aromatase inhibitor (AI) (post menopausal)	40	Ignatiadis (7), Dawson (8)
Luminal B	ER+/PR+/HER2+/Ki67	Tamoxifen (pre or post menopausal)	Aromatase inhibitor (AI) (post menopausal)	20	Ades (9)
HER2**+**	±ER+/HER2+	Trastuzumab, pertuzumab	Lapatinib	10	Baselga (10), Zhang (11)
Triple negative (TNBC)	EGFR/Notch-1/PI3K/-PTEN/- claudins/Hsp90/Ras	Molecular targeted therapies depending on subtype combined with chemotherapy, surgery, radiotherapy	Chemotherapy, surgery, radiotherapy	10–20	Lehmann (12)

## HER/HER Pathway

The HER/HER family is made up of four structurally related receptor tyrosine kinases (RTKs) with the EGFR as the founding member of the family. In humans, these include: HER1 (EGFR, HER1), HER2 (Neu, HER2), HER3 (HER3), and HER4 (HER4). *HER2*, the gene symbol, is derived from a homologous viral oncogene, Erythroblastic Leukemia Viral Oncogene, and has the official name: V-Erb-B2 Avian Erythroblastic Leukemia Viral Oncogene Homolog or HER2 accordingly (16).

**Figure 1:**
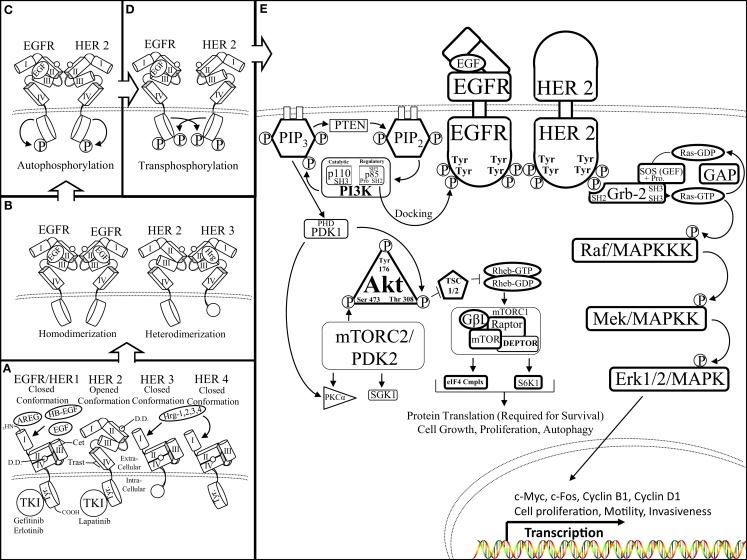
**The HER receptor and pathway: (A)** there are four members of the HER family of RTKs: EGFR/HER1, HER2, 3, and 4. Each HER receptor is composed of four functional extracellular domains (domain I, II, III, and IV) as well as a tyrosine rich (Tyr) intracellular domain. Epidermal growth factor (EGF), amphiregulin (AREG), heparin binding EGF (HB-EGF), and heregulin 1, 2, 3, 4 growth factor-ligands bind to domains II and III of EGFR/HER1 and HER3, HER4, respectively. Growth factor-ligand binding initiates HER conformational rearrangement causing exposure of the dimerization domain (DD), which is sandwiched between domains II and IV, as well as allowing the closed conformation of the HER receptor (portrayed by EGFR/HER1, HER3, 4) to assume an open conformation (displayed by HER2). It is important to note the truncated intracellular domain of HER3, which lacks kinase activity therefore, the HER3 receptor is the kinase dead member of the HER family of RTKs. **(B)** Ligand-mediated exposure of the dimerization domain enables the binding of two like HER receptors to form a homo-dimer (left, EGFR–EGFR) or two different HER receptors to form a hetero-dimer (right, HER2–HER3). It is important to note that the HER2 receptor is in a fixed, open conformation and does not require ligand binding to dimerize with a ligand bound HER receptor. **(C)** Upon HER dimerization, the HER receptors undergo auto-phosphorylation of their intracellular tyrosine residues. Auto-phosphorylation of the HER receptors primes the receptors to transphosphorylate the tyrosine residues of their binding partners **(D)** in preparation to initiate activation of downstream targets **(E)** activated HER receptors initiate the AKT (left) and mitogen activated protein kinase (MAPK) (right) pathways through a series of residue targeted phosphorylations, or a phosphorylation cascade. The Src homology domain 2 (SH2) of p85 (regulatory domain of PI3K) docks to the phosphotyrosine residue of the activated HER receptor (EGFR). p85 docking to EGFR enables Src homology domain 3 (SH3) of p110 (catalytic domain of PI3K) to join the proline rich region (Pro) of p85 to initiate PI3K 3′ phosphorylation of phosphatidylinositol (4, 5)-bisphosphate (PIP_2_) transforming PIP_2_ into phosphatidylinositol (3, 4, 5)-triphosphate (PIP_3_). PIP_3_ phosphorylation is aided by the Plekstrin homology domain (PHD) within the catalytic domain of phosphoinositide dependent kinase-1 (PDK1). The lipid binding PH domain recruits AKT to the plasma membrane where it docks to PIP_3_ as the physical interactions and activations of PI3K, PTEN, PDK1, as well as AKT occurs at the surface of the cell. PDK1 is able to phosphorylate AKT at the Thr 308 residue as well as aid in PKCa activation. AKT phosphorylation disrupts the formation of the tuberous sclerosis 1/2 (TSC 1/2) dimer, inhibits hydrolyzation of the GTP binding protein, Ras homolog enriched in brain (Rheb), to activate the mammalian target of rapamycin complex 1 (mTORC1) complex as well as the mTORC2 complex. The mTORC1 complex is composed of: G protein beta subunit-like (GβL), the metabolically sensitive regulatory-associated protein of mTOR (Rptor), DEP domain-containing mTOR-interacting protein (DEPTOR), and the serine/threonine kinase mammalian target of rapamycin (mTOR). The fully formed mTORC1 complex is able to promote cell growth, proliferation, and autophagy by directing protein translation by mediating the ability of Rptor to recruit the formation of the eukaryotic initiation factor 4F complex [eIF4F (eIF4E, G, B, etc.)] and ribosomal protein S6 kinase beta-1 (S6K1). The mTORC2 complex is also involved in cellular metabolism but mainly facilitates fluctuations in cytoskeletal formation and degradation throughout the cell via serum and glucocorticoid-regulated kinase 1 (SGK1), for instance. In a similar fashion, the SH2 domain of growth factor receptor bound-2 (Grb2) protein docks to the phosphotyrosine residue of the activated HER receptor (HER2). SH2 interaction with the Grb2 scaffold protein initiates the binding of two SH3 domains to the proline rich region of the son of sevenless (SOS) protein. SOS is a guanine-nucleotide exchange factor (GEF) that acts on Ras-GTPases (Ras-GDP) (rat sarcoma). SOS facilitates the unbinding of guanosine diphosphate (GDP) from Ras, there by destabilizing Ras and enabling guanosine triphosphate (GTP) binding to form Ras-GTP, and initiate the MAPK phosphorylation cascade. A GTPase activating protein (GAP) hydrolyzes Ras bound GTP (Ras-GTP), forming GDP, and halting downstream phosphorylation of the MAPK pathway. Ras activation begins the MAPK phosphorylation cascade, which is a series of serine/threoinine specific protein kinases occurring on: rapidly accelerating fibrosarcoma/mitogen activated protein kinase kinase kinase (Raf/MAPKKK), mitogen/extracellular signal-related kinase/mitogen activated protein kinase kinase (MEK/MAPKK), and extracellular signal-related kinase 1/2/mitogen activated protein kinase (Erk1/2/MAPK). Activation of the MAPK pathway initiates the transcription of a host of genes including: c-Myc, c-Fos, and cyclins to orchestrate cell motility, invasiveness, and proliferation.

Activation of the HER/HER family of receptors requires binding of a soluble, growth factor-ligand located in the intracellular cavity of the receptor that triggers receptor dimerization and phosphorylation, and activation of downstream pathways to elicit an appropriate response inside of the cell to the environmental stimuli outside of the cell. EGFR is activated by growth factor-ligands such as epidermal growth factor (EGF), Heparin binding EGF (HB-EGF), amphiregulin (AREG), or transforming growth factor alpha (TGF-α). In contrast, HER3 and HER4 are activated by the heregulin (neuregulin) family of growth factors (Hrg/Nrg 1, 2, 3, 4) (Figure 1A). Each HER/HER receptor, upon activation by growth factor binding, initiates hetero-dimerization or homo-dimerization of HER/HER receptors (Figure 1B). HER/HER receptor(s) dimerization stimulates auto-phosphorylation, followed by trans-phosphorylation of the receptor partner. HER/HER phosphorylation occurs at specific tyrosine residues within the intracellular domain of the HER/HER receptors. The phosphorylation of HER/HER tyrosine residues categorizes the HER/HER family RTKs (Figures 1C,D). These phosphorylated tyrosine residues recruit adaptor proteins such as Grb2 and the p85 subunit of the PI3K complex, which elicit the activation of several downstream pathways such as Protein Kinase B (AKT/PKB) and the mitogen activated protein kinase (MAPK) pathways (Figure 1E). HER/HER-mediated activation of downstream pathways enables a growth factor to elicit proliferation, survival, or migration of the cancer cell (Reviewed in Bublil and Kopan) (17, 18).

A significant number of ligand–HER receptor combinations are engaged to maintain normal cell physiology during development and throughout adult life. The mechanism of HER activation is controlled by ectodomain shedding in which proteolysis is mediated by a matrix metalloproteinase (MMP9 or ADAM12) that cleaves the ligand growth factors from the surface of the cell, near the transmembrane domain, to release the soluble ligand into the extracellular environment (19). All four members of the HER family of RTKs share structural similarities, but they have specific features that confer unique regulatory characteristics. Unlike EGFR, the extracellular region of HER2 resembles a ligand activated or fixed state, making HER2 an orphan receptor, thus not needing to bind ligands for its activation (20, 21). HER3 does not possess kinase activity and can only potentiate downstream signaling when dimerized with another HER receptor. The fixed state of HER2 makes it the preferred dimerization partner of the other HER receptors, particularly HER3. In addition, HER2 has been shown to dimerize with other RTKs, such as the Insulin-like Growth Factor-1 Receptor (IGF-1R) (22). HER2 dimerization enables HER receptors to broaden their ligand binding specificity as the HER ligands have non-overlapping functions, which restrict their binding to specific HER receptors (23–25). For example, only one EGF ligand is needed for downstream pathway activation when bound to EGFR and hetero-dimerized with HER2.

Epidermal growth factor receptor overexpression, or constitutive activation, has been implicated in the progression of a variety of cancers including lung, head and neck, colon, brain, and breast cancer by promoting tumor angiogenesis and metastasis (10, 26–31). EGFR inhibitors such as gefitinib (32) and erlotinib are tyrosine kinase inhibitors (TKIs). TKIs have been able to reduce EGFR oncogenic activity in some cases (33). Anti-EGFR monoclonal antibodies, such as cetuximab, bind to the extracellular domain of EGFR to inhibit growth factor binding and subsequent activation of downstream pathways (34).

HER2’s oncogenic activity confers a strong proliferative advantage to tumor cells, which includes increased tumor size, lymph node invasion, aneuploidy, percentage of cells in S-phase, and tumor grade resulting in an overall increased aggressiveness of the tumor (35–37). HER2 overexpression has been implicated in several human solid tumors including breast cancer in which a majority of the HER2 oncogenic activity, and its inhibition, has been studied. *HER2* gene amplification and subsequent HER2 protein overexpression occurs in 15–25% of DCIS (38) and invasive forms of HER2+ breast cancer. HER2 has been shown to increase p53 expression through an unknown mechanism in skin squamous-cell carcinoma (39). HER2 induction of p53 expression in HER2+ breast cancer cells may attribute to changes in Cyclin Dependent Kinase (CDK)/cyclin activity to promote cell division. Targeted anti-HER2 therapy includes the use of the antibody, trastuzumab, as well as TKIs, such as lapatinib. Trastuzumab is a humanized monoclonal antibody that binds to domain IV of the HER2 extracellular region causing reduced activation of the HER2 receptor (Figure 1A). Both anti-HER2 targeted therapies are potent inhibitors of HER2 activation in HER2+ tumors causing reduced disease progression (40). TKIs such as gefitinib and lapatinib are not strictly targeted to the tyrosine kinase activity of EGFR or HER2 alone but can affect the activity of both HER receptors.

An important trait of the HER family of receptors is the activation of the AKT/PKB and the MAPK pathways via HER phosphorylation. HER-mediated activation of PI3K causes the phosphorylation of the serine/threonine-specific protein kinase, AKT, which in turn activates mammalian Target of Rapamycin (mTOR). mTOR is comprised of two complexes: mTORC1 and mTORC2 and this review will focus on the ability of mTORC1 to induce protein synthesis that stimulates cell proliferation, migration, and metabolism (Reviewed in Altomore) (41). Many components of the AKT pathway have been implicated in tumorigenesis events such as the oncogenic activity of PI3K and the loss of the tumor suppressive activity of protein “Phosphatase and tensin homolog deleted on chromosome TEN (PTEN)”. These mutations have been suggested to drive cancer growth. Due to AKT’s role in a variety of human solid tumors and hematological malignancies, several therapies have been developed to target components of the AKT pathway to reduce tumor survival such as: Wortmannin and LY294002, which are reversible and non-reversible inhibitors of PI3K, respectively. HER activation of the MAPK pathway via docking of the Grb2 adaptor protein to HER phosphorylated tyrosine residue induces a Ras-mediated phosphorylation cascade resulting in the transcription of a number of genes that promote cell proliferation, survival, and cell migration (reviewed in Hynes) (42). Similar to the AKT pathway, several components of the MAPK pathway are involved in promoting tumorigenesis making these two pathways central nodes where activating mutations are known to trigger tumorigenesis and metastasis. Several inhibitors were designed to target components of the MAPK and mTOR pathways such as: U0126 and rapamycin, which inhibit Mitogen/Extracellular signal-related Kinase/Mitogen Activated Protein Kinase Kinase (MEK/MAPKK) and mTOR, respectively. EGFR or HER2-mediated stimulation of cell proliferation and survival pathways can cause cancer cells to become dependent on these pathways, or “addicted,” to EGFR or HER2 expression to maintain tumor growth and survival (43).

Unfortunately, cancers become resistant to many types of therapies that specifically target EGFR, HER2, or components of their downstream pathways. Resistance to these targeted treatments leads to tumor recurrence and in some cases a more aggressive cancer, ultimately ending in death of the patient. Cancer cells can adapt to treatments using a variety of mutations that enable the cancer cell to overcome targeted therapies and enable them to propagate under such conditions. It has come to light that cancer cells are able to become resistant to therapies by recruiting the use of a similar, compensatory pathway that continue downstream activation of protein synthesis, i.e., the AKT pathway, and/or transcriptional up-regulation of survival genes mediated by the MAPK pathway. Using compensatory pathways to divert dependence from one receptor to another is referred to as crosstalk, or bidirectional communication, between two pathways to allow continued cancer cell growth and survival. Extensive research has been done in G-Protein Coupled Receptor (GPCR) crosstalk with EGFR/HER2 as some GPCR agonists such as Lysophosphatidic acid (LPA), carbachol (muscarini acetylcholine inhibitor), and thrombin are known to be able to increase HER activation in two different ways (reviewed in Bray) (44). The first consists of a GPCR-dependent increase in the ectodomain shedding of HER ligands. The second phenomenon includes GPCR activation of c-Src to mediate phosphorylation of HER tyrosine residues. HER activation via GCPR may be required for GPCR-mediated mitogenic activity via the MAPK pathway (45). Both HER–MAPK pathway initiation, as well as activation of number of steroid hormones, can trigger the transcription of HER ligands and evidently initiate a positive feedback loop. Mechanisms of crosstalk between the HER family of RTKs and several other pathways including the: Wnt/β-catenin (46), TNFα/IKK/NK-κB (47, 48), and Notch pathway (49, 50) have been under thorough investigation for several years. Interesting findings have been made, which describe the novel mechanisms of HER–Notch bidirectional crosstalk. Recent data illustrate how these new mechanisms of crosstalk have been implicated in treatment resistance, mechanisms of Epithelial to Mesenchymal Transitioning (EMT), as well as the propagation and survival of BCSCs.

## Notch Pathway

**Figure 2:**
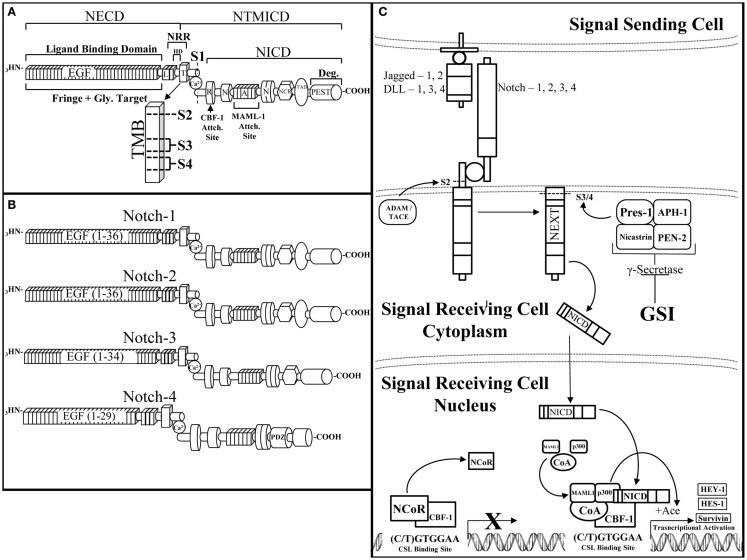
**The Notch receptor and pathway**. **(A)** The Notch receptor is composed of several domains that can be divided between the extracellular domain (NECD) and the transmembrane and intracellular domain (NTMICD). The NECD begins at the N-terminus (NH_3_) end of the Notch receptor and contains ligand binding epidermal growth factor-like (EGF) repeats and the negative regulatory region (NRR). Fringe proteins target the EGF repeats for glycosylation, which can augment their ligand binding ability to the Notch receptor. The NRR is composed of Lin12-Notch repeats (L) and the heterodimerization domain (HD), which aid in maintaining a closed conformation of the Notch receptor until ligand binding. The NTMICD contains: the transmembrane domain (T, TMB), the RBP-Jκ associating module (R) or RAM domain, one of two nuclear localization signals (N), the ankyrin domain, a second nuclear localization signal, the cysteine response region (NCR), the transactivating domain (TAD), and a proline/glutamic acid/serine/threonine-rich motif (PEST) at the C-terminal (COOH) end of the Notch receptor. Ligand bound Notch is cleaved at a series of scissile sites (S1, 2, 3, 4), which are represented just outside of (S1) and inside the transmembrane domain (S2, 3, 4) (TMB enlargement). Furin-like convertase cleaves immature Notch at S1 to create the mature, heterodimeric Notch receptor, which is stabilized by a non-covalently bound calcium ion (Ca). The RAM domain facilitates core binding factor-1 (CBF-1) binding, the ankyrin domain binds mammal-like mastermind-1 (MAML-1) and p300 (p300), which are necessary components of the Notch transcriptional activating complex. The PEST domain is responsible for degradation of the Notch intracellular domain (NICD). **(B)**. There are four Notch receptor paralogs, each similar in overall composition but with slight structural differences that facilitate their diverse attributes. From top to bottom are Notch-1, -2, -3, and -4. Notch-1 is the most studied Notch receptor containing all of the above mentioned domains with 36 EGF-like repeats. Notch-2 is similar to Notch-1 except for differences in its EGF-like repeats as well as the ability of Notch-2 to bind to Jagged-1 upon fringe modification (118). Notch-3 has a slightly truncated EGF-like domain with 34 repeats and lacks a transactivating domain. Notch-4 is the smallest of the Notch receptor paralogs with 29 EGF-like repeats, a shorter NICD due to a lack of a transactivating domain and cysteine response region, as well as the addition of a PDZ-Domain [Post-synaptic Density Protein (PSD)-95] (PDZ) that plays a critical role in proper neuronal development (119). **(C)** The Notch pathway is activated by a Notch-ligand (jagged-1, -2, DLL-1, 3, 4) expressed on a signal sending cell binding to a Notch receptor (Notch 1, 2, 3, 4) on a signal receiving cell. Ligand bound Notch undergoes a series of cleavages, first by a disintigrin and metalloproteinase/tumor necrosis factor alpha converting enzyme (ADAM/TACE) at the S2 cleavage site to form the Notch extracellular truncation (NEXT) fragment. The NEXT fragment is targeted by the γ-Secretase complex for cleavage. The γ-secretase complex is composed of nicastrin, anterior PHarynx-defective 1 (APH-1), presenilin ENhancer 2 (PEN-2), and the active component of the complex, presenilin-1. γ-Secretase is inhibited by γ-secretase inhibitors (GSIs), which prevent Notch cleavage and transcriptional activation. Proper cleavage of the Notch receptor releases NICD, allowing NICD to travel to and enter the nucleus where NICD binds to promoter bound CBF-1, allowing CBF-1 to release negative co-regulators (NCoR) and recruit transcriptional co-activators. NICD-mediated transcription cannot occur without the recruitment of mammalian mastermind-like-1 (MAML-1) and the histone acetyltransferase, p300, to unwind the compressed DNA allowing transcription of the Notch targeted genes. Notch activates the transcription of canonical target genes such as: hairy enhancer of split-1 (HES-1), hairy/enhancer of split with an YRPW motif-1 (HEY-1), and non-canonical Notch target genes such as survivin.

The Notch receptor is a single-pass, type I, transmembrane receptor (Reviewed by Kopan et al.) (Figure 2A) (51). A mature Notch receptor is formed as a hetero-dimer, composed of two domains bound by a non-covalent bond, typically mediated by calcium (Ca^2+^). Upon maturation, the full length Notch polypeptide is cleaved at the S1 site, within the Golgi-apparatus by a furin-like convertase, into two domains: the Notch extracellular (NECD) and transmembrane/intracellular (NTMICD). The extracellular portion of the Notch receptor contains a series of EGF-like repeats that mediate ligand interactions from a neighboring cell. The EGF repeats are followed by the Negative Regulatory Region (NRR) that prevents ligand-independent activation of the Notch receptor. There are five known Notch-ligands: Jagged-1 and -2 (Jag-1, -2), Delta-Like-1, -3, and -4 (DLL-1, -3, -4) that bind to and activate the Notch receptor when two neighboring cells are in close proximity to each other (Figure 2C). The affinity of a Notch-ligand to its Notch receptor can be attenuated by Fringe glycosyltransferases (Lunatic, Manic, or Radical) by adding *N*-acetylglucosamine moieties to the NECD region. The intracellular domain of the Notch receptor contains the active portion of the Notch receptor, the Notch Intracellular Domain (NICD), which is composed of the: RBP-Jκ associating module (RAM) domain, ankyrin domain, Cysteine Response region (NCR), Transactivation Domain (TAD), proline/glutamic acid/serine/threonine-rich motifs (PEST domain), and a pair of Nuclear Localization Sequences (NLS) that sandwich the Ankyrin domain to direct cleaved NICD to the nucleus enabling NICD to initiate transcriptional activation of target genes. Activation of the Notch pathway requires a series of events, which begins with the Notch receptor on a signal receiving cell binding to a Notch-ligand on the neighboring signal sending cell. Consequently, this ligation of ligand to receptor is required for removal of the inhibitory NECD from the NTMICD causing two subsequent cleavages at S2 mediated by ADAM10/17 and S3/4 mediated by the γ-secretase complex. The ADAM and γ-secretase targeted cleavage sites are buried within the transmembrane domain of the Notch Extracellular Truncation (NEXT) fragment. Cleavage by the γ-secretase complex releases the NICD portion of the Notch receptor (Figure 2A). NICD shuttles to the nucleus where it acts as a Recombining Binding Protein-Jκ/Core Binding Factor-1 (RBP-Jκ/CBF-1) mediated transcriptional activator of target genes. NICD binds to CBF-1, which is already bound to DNA, enabling the release of negative co-regulatory proteins such as C-terminal-Binding Protein 1 (CtBP1) and SMRT/HDAC-1-Associated Repressor Protein (SHARP) and the recruitment of co-activating proteins such as Mammal Mastermind-like-1 (MAML-1) and the Histone Acetyltransferase (HAT), p300, to CBF-1, to form the Notch transcriptional activating complex. Canonical Notch target genes are primarily basic helix-loop-helix transcriptional repressors including, Hairy/Enhancer of Split-1 (HES-1-5) and Hairy/Enhancer of split with a unique YRPW motif-1 (HEY-1, HEY-L) (Figure 2C). There are four Notch paralogs (Notch-1, -2, -3, -4) each with quite different structures and possibly synergistic and/or counteracting functions (Figure 2B).

The Notch pathway is most commonly associated with lateral inhibition in which the Notch-ligand, Delta, if expressed in one cell can inhibit Delta expression by a neighboring cell. In a cluster of undifferentiated cells, when one cell begins to develop into an epithelial cell, it signals its neighbors to do the same. Initially, an undifferentiated cell expresses both the Notch receptor and the Delta ligand. Activation of the Notch receptor inhibits Delta production in the same cell. Through juxtacrine signaling, neighboring cells compete to produce Delta, resulting in a feedback loop that drives the two neighboring cells to assume different cell fates based on the number of Notch receptors or Delta ligands expressed by each cell. Notch signaling is used to establish a border between two different populations of cells such as stromal and progenitor cells. Boundary formation by oscillating Notch activity is most notable in somite formation in which the constant activation and inactivation of Notch-mediated transcriptional activity causes the segmented formation of somites in vertebrates (52–54). Notch is able to dictate cell lineage by asymmetrical inheritance of Notch regulators between two dividing, daughter cells, and is most evident during neurogenesis (55). Protein encoded by the gene *NUMB* (Numb Drosophila Homolog), is an inhibitor/negative regulator of Notch activity. Asymmetric distribution of Notch and Numb between two dividing cells determines if one daughter cell is a signal sending cell and if the other is a signal receiving cell. This uneven distribution of Notch and Notch regulators, Numb, is passed down through multiple cell divisions to maintain stem cell populations as well as influence cell lineage decisions (56). The culmination of the Notch receptor studies resulted in determining the Notch pathway and its ability to act as a form of short range communication between cells. Notch juxtacrine signaling has been found to be involved in a variety of processes, most notably in stem cell differentiation, self-renewal, and cell fate determination as well as cell proliferation, growth, and survival (Reviewed by Artavanis-Tsakonas) (57).

The Notch pathway has been referred to as the Notch-survival pathway due to promotion of cell growth and proliferation. One of the Notch gene targets responsible for regulation of cell longevity is called survivin. Survivin is an Inhibitor of Apoptosis Protein (IAP) and functions to down-regulate caspase 3, 7, and 9 apoptotic activity as well as regulate the cell cycle by interacting with spindle microtubules during mitosis (58). Cyclin Dependent Kinase-1 (CDK1) phosphorylates the threonine 34 residue of survivin to stabilize its protein structure (59, 60) and enabling survivin to escape degradation mediated by the X-linked IAP-X-linked Associating Factor-1 complex (XIAP-XAF1). The XIAP-XAF1 complex induces E3 ligase activity that targets survivin for ubiquitination and subsequent proteasomal degradation. Prolonged survivin expression has been associated with breast cancer cell survival via the MAPK/ERK and AKT/PI3K pathways (61). A correlation has been made between poor breast cancer prognosis and an increase in the expression of both Notch-1 and survivin in ER-breast cancer and has been described as the Notch-Survivin signaling axis (62).

There is a growing body of evidence that Notch up-regulation or mutation results in several events that enable breast cancer cells to: become resistant to targeted treatments, undergo EMT, metastasize, and promote BCSC survival, and self-renewal (63). Increased co-expression of Notch-1 and Jagged-1 has been associated with poor prognosis for women diagnosed with breast cancer (64). Notch-1, -3, or -4 have shown oncogenic activity in T cell acute lymphoblastic leukemia (T-ALL) (65), B-cell lymphoma (66), cervical (67), colon (68, 69), lung (70), and ovarian (71, 72) cancers while, surprisingly, Notch-2 has displayed tumor suppressive activity in some breast cancer subtypes (73–75). There are a number of studies characterizing Notch as a tumor suppressor in some myeloid malignancies (76, 77). Notch targeted treatments include γ-Secretase Inhibitors (GSIs), which inhibit proteolytic processing and subsequent NICD-mediated transcriptional activation by two different modes of action. First, transition state analog GSIs mimic the transition state of a substrate that is cleaved by γ-secretase thereby binding to the catalytically active subunit of γ-secretase, presenilin-1, to inhibit GSI-mediated cleavage of a substrate, such as NEXT (Figure 2C). Second, non-transition state analog GSIs are small molecule inhibitors that bind to the γ-secretase in an allosteric manner (regulate GS enzymatic activity by not binding to the active site of GS), to interfere with its ability to cleave and activate the Notch receptor (78) (Reviewed by Olsauskas-Kuprys) (79). There are several distinct GSIs (DAPT, MRK-003, Compound E) as well as the continued development of selective Notch inhibitors, γ-secretase modulators (PF-03084014), which are able to inhibit specific Notch paralogs (80). Continued research into the role of the Notch pathway in tumorigenesis has revealed novel crosstalk with the EGFR and HER2 receptors in a variety of solid tumors, including breast cancer.

## Notch–EGFR Crosstalk

Researchers have continued to elucidate crosstalk between Notch and EGFR in hopes to dissect the mechanism(s) by which this crosstalk occurs and to better comprehend how cancer cells use the Notch pathway to compensate for EGFR targeted inhibition. Crosstalk between the Notch and EGFR signaling pathways has been observed in the *Drosophila* eye and wing development in which the two pathways can promote or antagonize each other to select which phenotype is produced, depending on the developmental context (81–83). Research into the role of the Notch–EGFR crosstalk has since been observed in lung (70), skin (84), and brain (85) cancers. Current investigation of the bidirectional communication between EGFR and Notch led to its observation in non-small cell lung cancer by Xie et al. as a route of acquired resistance to EGFR targeted TKIs and development of the EMT phenotype via Notch-1 mediated down-regulation of E-cadherin (epithelial marker), as well as up-regulation of vimentin (mesenchymal marker), and Snail (mesenchymal marker) expression (86). Notch–EGFR crosstalk has been shown to increase MUC5AC expression (87) causing increased goblet cell secretion of mucin, a feature attributed to chronic airway inflammatory disease as well as a potential activator of inflammatory induced lung cancer. EGFR has been shown to inhibit expression of the *Notch-1* gene in a squamous-cell carcinoma model by up-regulating c-Jun, which in turn down-regulates p53, causing repression of Notch-1 transcription, and this form of Notch-1-EGFR crosstalk occurs via the MAPK/MEK/ERK pathway (84). These are just a few examples of Notch–EGFR crosstalk promoting drug resistance, EMT, and disease progression in various carcinomas.

Activation of the PI3K pathway by deletion or inactivation of PTEN or oncogenic mutation of PIK3CA has been observed in many types of cancer as well as being implicated in drug resistance. Down-regulation of PTEN and oncogenic mutation of PIK3CA compounded with EGFR over expression has been correlated with poor response to trastuzumab treatment as well as a poor clinical outcome in women with HER2+ breast cancer (88, 89). While PI3K is a downstream target of EGFR signaling, growing evidence suggests that Notch activates the PI3K pathway to promote survival. The regulation of the PI3K pathway by Notch has been shown by Palomero et al. Specifically, the transcriptional target of Notch-1, HES-1 has been shown to repress PTEN expression resulting in increased PI3K signaling in T-ALL (90). Furthermore, Jagged-1-mediated activation of Notch signaling was shown to increase the phosphorylation and thus the activation status of AKT1 in cervical cancer cell lines to promote EMT and increase cell motility (91). Previous studies have shown that inhibition of the PI3K pathway, using small molecule inhibitors that target AKT or the mTOR signal transduction pathway, are able to restore sensitivity to trastuzumab treatment (92). Similarly, Eichhorn et al. showed that the use of a dual PI3K and mTOR inhibitor, NVP-BEZ235, can reverse anti-EGFR/HER2 resistance in HER2+ breast cancers that express low PTEN or contain PIK3CA activating mutations (93). These results strongly indicate that both Notch and EGFR activate the PI3K pathway suggesting that the two pathways converge to promote proliferation and survival.

**Figure 3:**
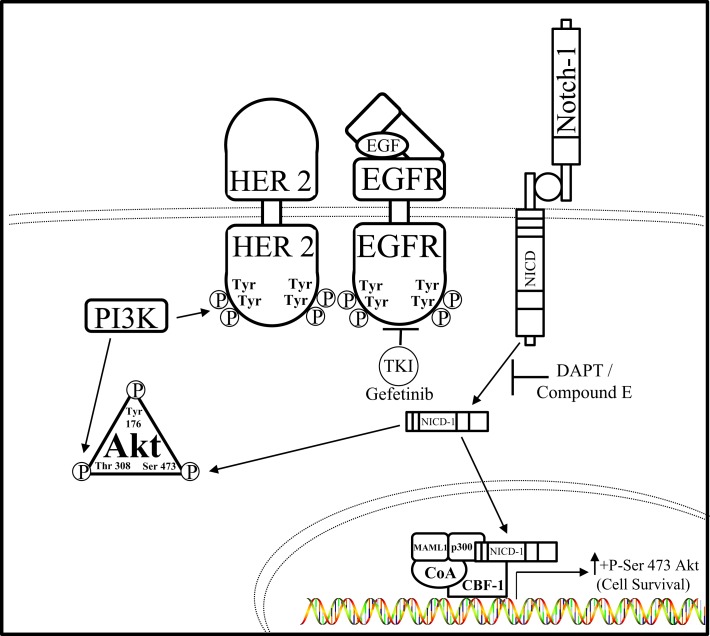
**Notch–EGFR crosstalk in TNBC**. Dong et al. ascertained a form of crosstalk between EGFR and Notch receptors that enabled resistance to EGFR targeted therapy, gefitinib, in TNBC cells. It is proposed that EGFR targeted resistance occurs by Notch-1-mediated activation of the AKT pathway. Notch–EGFR crosstalk facilitates an increase in serine-473 residue phosphorylation of AKT, which allows TNBC cells to survive EGFR targeted treatments. Dual inhibition of EGFR via the tyrosine kinase inhibitor (TKI) gefitinib and the Notch pathway by GSIs, N-[N- (3,5-difluorophenacetyl)-l-alanyl]-S-phenylglycine t-butyl ester (DAPT) *in vitro*, and compound E *in vivo*, are synergistically lethal to TNBC tumorigenesis.

Hyperactivation of both the Notch and EGFR pathways were observed in TNBC cells by Dong et al. leading them to hypothesize that Notch–EGFR crosstalk occurs in this aggressive breast cancer subtype (94). Inhibition of EGFR or Notch alone was insufficient to reduce TNBC tumor burden, leading to the possibility that these complementary pathways are capable of interacting with one another to confer resistance to EGFR or Notch targeted therapies, hence bidirectional crosstalk between the EGFR and Notch pathway. Therefore, combined Notch (DAPT, Compound E) and EGFR (gefitinib) inhibition caused a decrease in TNBC cell proliferation and an increase in TNBC cell death both *in vitro* and *in vivo* indicating that a synthetic lethal relationship exists between the EGFR and Notch pathways. Furthermore, the AKT pathway has been shown to promote resistance to Notch inhibition (95, 96) as well as resistance to EGFR inhibition (97–99). Inhibition of EGFR or Notch receptors alone has little effect on the activity of AKT, but dual inhibition showed dramatically reduced level of serine-473 phosphorylation of AKT *in vitro*. Overexpression of NICD1 in the EGFR expressing TNBC cell line, HCC1806, caused resistance to combined DAPT and gefitinib treatment indicating that the Notch–AKT pathway confers resistance to combined EGFR and γ-secretase inhibition in this TNBC model. Unfortunately, all TNBCs do not have the same mutation profiles and some lack overexpression of EGFR. Importantly, a large group of TNBC malignancies with low EGFR expression are not affected by anti-EGFR or anti-Notch treatments, as seen by *in vitro* studies performed in MDA-MB-231 with a K-Ras mutation (100, 101) (Figure 3). These results indicate a bidirectional crosstalk between Notch and EGFR occurs in breast cancer. Clear evidence provided shows that both Notch and EGFR pathways are used by TNBC cells to escape targeted inhibition by compensatory activation of the Notch pathway. Compensatory Notch pathway activation maintains MAPK and/or AKT downstream signaling pathways making the breast cancer cell resistant to EGFR targeted treatment. Preclinical studies have combined EGFR and HER2 inhibitors with MAPK/MEK or AKT/PI3K/mTOR inhibitors to reduce resistance to EGFR/HER2 TKIs (92, 93, 102). One mechanism that has been proposed is that EGFR regulates the Notch pathway through the repression of the global transcriptional co-repressor, Goucho/TLE (Transducer like Enhancer of split) (103) to attenuate Notch-mediated gene activation and promote growth of cancer cells. EGFR targeted inhibition alone has been an ineffective treatment strategy for TNBC and understanding the mechanisms of EGFR resistance as well as improving therapeutic strategies for TNBC are critical areas of research to improve survival rates for these women. Dual targeting of the EGFR and Notch pathways may become a viable treatment option for women diagnosed with EGFR over expressing TNBC.

## Notch–HER2 Crosstalk

Notch–HER2 crosstalk was first identified in breast cancer cells by Osipo et al. who observed that HER2+ breast cancer cells had low Notch-1 expression and activation. Upon treatment with an anti-HER2 therapy (trastuzumab), or observing HER2+ cells with an acquired resistance to an anti-HER2 therapy, it was noted that Notch-1 expression and Notch transcription of canonical target genes increased (50). This important research described siRNA knockdown or GSI-mediated inhibition of Notch-1 enhanced trastuzumab sensitivity and reversed resistance to trastuzumab treatment *in vitro*. These findings led to the conclusion that HER2 inhibition increased Notch-1 activity in a compensatory manner to promote survival and resistance of HER2+ breast cancer cells. Combinatorial targeted treatment of Notch and HER2 signaling pathways not only inhibited HER2+ tumor formation but more importantly recurrence (104).

**Figure 4:**
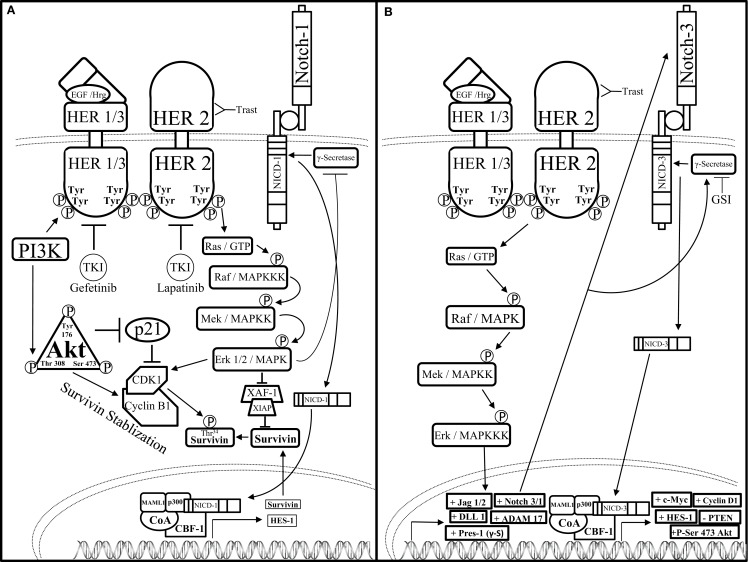
**Notch–HER2 crosstalk in HER2+ BC (A)**. Ju et al. determined an intricate form of crosstalk between HER2 and Notch-1 in HER2+ breast cancer. HER2+ breast cancer cells exhibit an increase in survivin protein expression concomitantly with a decrease in survivin mRNA expression. HER2 promotes activation of the AKT pathway, which threonine 308 phosphorylated AKT can inhibit p21-mediated inhibition of cyclin dependent kinase-1 (CDK1) dimerization to cyclin B1. Concomitantly, HER2-mediated activation of the MAPK pathway enables Erk-mediated CDK1-Cyclin B1 dimerization as well as inhibition of ubiquitination and proteasomal degradation of survivin by restricting the formation of the X-linked Inhibitor of apoptosis protein-X-linked associating factor-1(XIAP-XAF-1) complex. Together, AKT and Erk promote dimerization of CDK1 to Cyclin B1, which stabilizes survivin by phosphorylating the threonine 34 residue (Thr^34^) of the survivin protein. Increased survivin stabilization causes an increase in survivin expression. Survivin mRNA is reduced by Erk-mediated inhibition of γ-secretase, which in turn inhibits Notch receptor-mediated transcriptional activation. **(B)** Pradeep et al. established an interesting form of crosstalk between the HER2 and Notch-3 receptors in HER2+ DCIS. HER2 up-regulates the transcription of the Notch pathway components: jagged-1, -2 (Jag-1/2), DLL-1, ADAM17, presenilin-1 (Pres-1), as well as Notch-3 and Notch-1 (Notch-3/1) through the MAPK pathway. Increased transcription of the Notch pathway components causes Notch-mediated up-regulation of c-Myc, Cyclin D1, phosphorylation of Serine-473 of Atk (+P-Ser 473 AKT), and down-regulation of the tumor suppressor, PTEN. Combined GSI and trastuzumab (Trast) treatment reduce HER2+ DCIS cell survival and invasiveness.

One mechanism by which HER2 restricts Notch signaling was identified by Ju et al. These investigators showed that HER2 overexpressing breast cancer cells activate the ERK pathway to block the activity of the γ-secretase complex thus resulting in reduced levels of NICD1. Interestingly, these investigators also showed that Notch-1 is a transcriptional repressor of the *Survivin* gene, a potent anti-apoptotic gene that is regulated at the protein level by the E_3_ ligase complex, XIAF1:XIAP. Specifically, Ju et al. showed that the HER2–ERK axis represses XIAF1, which is required to ubiquitylate the survivin protein. This resulted in increased survivin protein stability to promote survival of HER2+ breast cancer cells. Therefore, these results suggest that HER2 signaling via ERK inhibits Notch-1 cleavage thus limiting the formation of NICD1 and stabilizes the survivin protein (105) (Figure 4).

Conversely, crosstalk between HER2 and Notch could be stimulatory rather than inhibitory. One of the first studies to implicate crosstalk between Notch-1 and HER2 was published by Chen et al. where they showed that the HER2 promoter contained a putative CBF-1 binding site (106). Specifically, these investigators demonstrated that overexpression of NICD1 induced transcription of HER2 suggesting that Notch-1 positively regulates HER2 expression. Moreover, Notch has been shown to regulate HER2 expression in BCSCs thereby controlling the survival and differentiation of BCSCs (107), which have been implicated in promoting breast cancer cell drug resistance, metastasis, EMT, as well as tumor recurrence and growth. Furthermore, Pradeep et al. demonstrated that HER2 overexpression in MCF-10A-DCIS cells increased Notch-3-mediated transcription of Notch target genes to promote a more malignant phenotype (108). In the HER2+ DCIS cell model, Notch-3 nuclear localization was increased along with ADAM17, presenilin-1, Jag-1, DLL-1, and Notch-1, all components of the Notch pathway. Knock down of Notch-3 reduced HER2 + DCIS cell proliferation, spheroid formation, and luminal spheroid filling indicating that HER2-mediated up-regulation of Notch-3 promoted HER2+ DCIS cell proliferation and survival. Findings by Pradeep et al. imply that HER2-mediated activation of Notch-3 is necessary during the early steps of mammary tumorigenesis and targeted inhibition of these pathways can reduce early tumor progression in DCIS-HER2+ cancers with Notch-3 overexpression.

**Figure 5:**
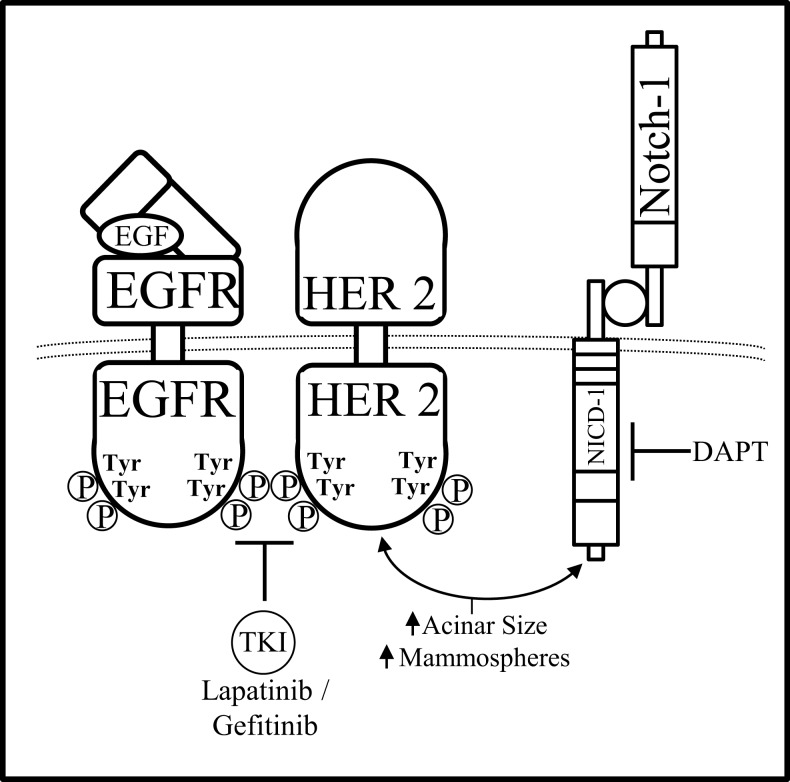
**Notch–HER2 crosstalk in HER2+ DCIS**. Farnie et al. discerned a form of Notch–EGFR/HER2 bidirectional communication in an *in vitro* DCIS breast cancer model that promoted acinar size and mammosphere (BCSC) formation. Dual inhibition of the Notch–EGFR/HER2 receptors using DAPT (GSI) and gefitinib/lapatinib, respectfully, caused decreased acinar size and mammosphere formation while either inhibitor alone effected only acinar size or only mammosphere formation.

Combined inhibition of Notch and HER pathways, EGFR or HER2 via gefitinib or lapatinib, respectively has been investigated in a DCIS model by Farnie et al. to deduce the effects of dual inhibition of the Notch–EGFR/HER2 pathways on DCIS stem cells (109). Using a 3D culture system to assess luminal filling of DCIS acini as well as DCIS Cancer Stem Cell (CSC) activity, two DCIS cells lines, SUM225 (HER2−), and MCF10DCIS HER2 (HER2+), as well as human primary DCIS samples (HER2±) were studied. These two cell lines were treated with GSI (DAPT) and/or anti-HER1/2 (gefitinib or lapatinib) therapies to deduce the role of Notch–EGFR/HER2 crosstalk in HER± DCIS breast cancer. Anti-Notch treatment reduced mammosphere formation and acini size in the HER2+ cells yet had no effect on the HER2− cells. Anti-EGFR/HER2 treatment had the opposite effect by reducing mammosphere formation and acini size on HER2− cells with no effect on the HER2+ cells. Interestingly, combined inhibition of the Notch and EGFR/HER2 receptors reduced both acini size and mammosphere formation regardless of HER2 expression indicating that the crosstalk between Notch and EGFR/HER2 receptors maintains BCSC survival and self-renewal (Figure 5). In addition to these studies, others have shown that HER2 may facilitate increased expression of components of the Notch pathway such as Notch-ligands (Jag-1, Jag-2, DLL-1) or Notch activating metalloproteinases (ADAM, presenilin-1).

Notch–HER crosstalk can involve upstream and/or downstream components of either pathway. Recent data have shown Notch-dependent up-regulation of the ADAM12 metalloproteinase causes an increase in ectodomain shedding of HB-EGF, an EGFR growth factor, in head and neck squamous-cell carcinoma cells under hypoxic conditions. Notch-mediated release of HB-EGF causes the formation of invadopodia to aid in cancer cell invasion (110). Notch–EGFR crosstalk has been implicated as a paracrine mediator of estrogen to promote ER−/ER low BCSC survival and proliferation within ER+ breast cancer cell lines and patient samples (111). Recent data have shown that estrogen increases ER+ bulk cell (Non-BCSC) production of EGFR, FGFR, and Notch-ligands, which are able to regulate ER−/ER low BCSC differentiation and expansion via the EGFR/MAPK/ERK, FGF/FGFR/Tbx3, and DLL-Jag/Notch/Pea3 pathways. Estrogen-driven BCSC regulation can be inhibited by blocking estrogen using tamoxifen, EGFR using gefitinib, or Notch using a GSI to attenuate BCSC survival and early progenitor cell differentiation, and possibly de-differentiation, respectively. Crosstalk between EGFR, FGFR, and Notch pathways are responsible for estrogen induced changes in the ER−/ER low BCSC population and may play a role in endocrine resistance as well as offer suitable targets for the treatment of ER+ breast tumors. Combinatorial drug treatments simultaneously targeting Notch and EGFR receptors continue to be assessed to reduce the occurrence of chemo-resistance in cancers such as glioblastoma. Sooman et al. treated six glioblastoma cell lines with drugs such as imatinib (TKI), camptothecin, or temozolomide, and measured gene expression using microarray to identify molecular pathways associated with drug resistance, then combined the cytotoxic treatments with targeted inhibitors to determine optimal combinatorial treatments for glioblastoma (112). Synergistic effects of camptothecin with gefitinib or NSC 23766 [ras-related C3 botulinum toxin substrate 1 inhibitor (RAC1)], as well as imatinib with DAPT or NSC 23766 and additive effects of temozolomide with gefitinib or PF-573228 (focal kinase inhibitor) to sensitize glioblastoma cells to cytotoxic chemotherapy causing cancer cell death and growth arrest. Careful consideration of the effect of these drug combinations on non-cancerous epithelial cells was taken and the analyses presented potential gene expression signatures that correlate to drug sensitivity and could be used as predictive factors for a patient’s treatment response.

Most of the studies to date have focused on mechanisms of crosstalk between Notch and the PI3K pathway. Specifically, Ma et al. showed that hyperactive mTOR signaling correlates with increased Notch signaling in poorly differentiated breast cancers and is also associated with a poor clinical outcome in women with mTOR/Notch over expressing tumors (113). A variety of cancer cell lines, including ER+ MCF7 and triple negative MDA-MB-486 expresses elevated mTOR signaling and Notch activity. These cells were used to ascertain the correlation between the two hyperactive pathways. Ma et al. examined this correlation to find that RTK activation of the PI3K/mTORC1 pathway caused up-regulation of several new mTORC1 effectors: Signal Transducer and Activator of Transcription 3 (STAT3), p63 (a member of the p53 family), Jagged-1, Notch, and HES-1. Aberrant RTK/PI3K/AKT/mTORC1 signaling caused an increase in STAT3, which in turn increased p63 transcription. Elevated p63 increased Jagged-1-mediated Notch activation and thus HES1 expression. This study presents the mechanism by which the RTK/PI3K/AKT/mTOR pathway up-regulates the STAT3/p63/Jag-1/Notch signaling cascade to induce tumorigenesis. It has been reported that Notch is a positive regulator of the PI3K/AKT/mTOR pathway in T-ALL (106, 107) suggesting that mTOR and Notch may cooperate in a shared regulatory loop to influence cancer cell differentiation or proliferation in a dose dependent manner.

In another study by Mungamuri et al., increased Notch-1 expression and activity in cancer cells was shown to promote cell survival, proliferation, as well as chemo-resistance. Specifically, the investigators elucidated crosstalk between the Notch and PI3K pathway in several different cancer cell lines, including a chemoresistant breast cancer cell line (MCF7), with increased Notch-1 expression and signaling (96). Activated Notch-1 signaling was found to inhibit p53 activation by blocking nuclear localization as well as phosphorylation at Ser^15^, Ser^20^, and Ser^392^ residues. Blocking mTOR signaling using rapamycin treatment was shown to sensitize cancer cells to chemotherapy by preventing Notch-1-mediated down-regulation of p53. Conversely, up-regulation of the mTOR downstream target eukaryotic initiation factor 4E (eIF4E) caused chemo-resistance as well as inhibition of p53-mediated apoptosis. These findings also support the critical role of the PI3K/mTOR pathway on Notch-1-mediated regulation of apoptosis and drug resistance.

An intriguing mechanism of communication between the Notch and MAPK pathways has been elucidated by Izrailit et al. (114). Active Notch-1 expression in the TNBC cell line MDA-MB-231 was found to use the MAPK/ERK pathway to facilitate Jagged-1 up-regulation and subsequent Notch activation. The TGFβ pathway has also been implicated in regulating Jagged-1 expression to promote bone metastasis in breast cancer (115). Using high throughput screening, Izrailit et al. found that the pseudokinase Tribble-3 (TRB3) regulates activation of both the MAPK/ERK and TGFβ pathways. Knock down of TRB3 reduced MDA-MB-231 cell proliferation while Jagged-1 over expression in the TRB3 silenced cells rescued cell proliferation. Similar studies were done *in vivo* in which the MDA-MB-231 cells with TRB3 knock down had significantly smaller tumors than mice with without TRB3 knock down. These results indicate that TRB3 increases Jag-1 expression through the MAPK/ERK pathway and this is critical to MDA-MB-231 cell proliferation and tumorigenesis.

The studies in this review reveal communication between the Notch and HER pathways in breast cancer cells that enable the cell to compensate for the loss of one pathway with the potential compensatory increase in the other. This research is continuing to unveil multiple interactions between multiple pathways to discern the in depth complexity by which breast cancer cells are able to thrive under a variety of targeted or cytotoxic cancer treatments. Continued work in this area may shed light onto the intricacies of pathway to pathway communication that could be utilized in other forms of cancer and diseases. The Notch and HER pathways have overlapping functions that justify their compensatory relationship as both are able to promote cell survival, growth, and motility, but nuances of either pathway may be able to be teased out to further assess effects of compiling multiple targeted cancer treatments. For instance, the Notch pathway has also been associated with increased angiogenesis via DLL4, IL-1, or Leptin-mediated up-regulation of VEGF/VEGFR (116) as well as AKT/mTOR-mediated up-regulation of glucose metabolism via up-regulation of Glut 1 (117).

This review has summarized the communication mechanisms between the Notch and HER receptors with a particular focus on PI3K and MAPK signaling pathways. More studies are needed to uncover novel interactions, downstream pathway mutations, microRNA dysregulation, or epigenetic changes that may be critical to understanding how cancer cells move between pathways to create their own signaling circuit for survival. Further assessment of Notch–HER crosstalk in BCSC and stromal-cell communication could reveal differences in autocrine signaling to promote BCSC growth and survival. Continuing to determine mechanisms of action between critical oncogenic pathways such as Notch and HER may enable scientists to better diagnose and treat breast and other solid tumors using an increasingly specialized treatment plan that may preemptively avoid development of treatment resistance, CSC growth, and tumor recurrence.

## Conclusion

Notch–HER crosstalk continues to yield insight into the compensatory mechanisms used by breast and other cancer cells to propagate cell survival, growth, metastasis, as well as drug resistance. Combinatorial treatments targeting the HER and Notch pathway simultaneously have yielded positive results in attempts to combat acquired resistance to molecular targeted and cytotoxic therapies. The goal is ultimately, to prevent or reverse drug resistance, as well as providing gene signatures that could preemptively reveal potential resistance and sensitivity to combinatorial treatment plans, in women diagnosed with HER+ or ER+ breast cancer. Continued research will unravel mechanisms of Notch–HER crosstalk to further improve clinical response to emerging therapies as well as revitalize current therapies in hopes of continued improvement of treatment strategies for women with breast cancer and other solid tumors.

Each breast cancer subtype is represented in Table 1 as well as the molecular markers that each breast cancer subtype tends to over express. The most common first and second line treatment therapies for each subtype are included and it is important to note that these therapies change based on the patient’s family history, age, race, as well as tumor stage (invasiveness) and histological grade. The rates of breast cancer subtype occurrence are approximated in this table (1). Advances in genomic driven classification of breast cancer continue to evolve the subtypes, particularly in TNBC, which can be further divided into: basal-like 1, basal-like 2, immunomodulatory, mesenchymal-like, mesenchymal stem-like, and luminal androgen receptor (12). Typical treatment options are neoadjuvant, or adjuvant, meaning they include variations of chemotherapy and/or radiotherapy before or after surgery, respectively, in an effort to remove/eradicate as many tumor/cancer cells as possible.

## Conflict of Interest Statement

The authors declare that the research was conducted in the absence of any commercial or financial relationships that could be construed as a potential conflict of interest.

## References

[1] American Cancer Society. Breast Cancer Facts & Figures 2013-2014. Atlanta, GA (2013).

[2] Perou CM Surlie T Eisen M Van de Rijn M Rees C Pollack J Molecular portraits of human breast tumours. Nature (2000) 406(17):747–52 10.1038/35021093 10963602

[3] Hu Z Fan C Oh DS Marron JS He X Qaqish BF The molecular portraits of breast tumors are conserved across microarray platforms. BMC Genomics (2006) 7(1):96. 10.1186/1471-2164-7-96 16643655 PMC1468408

[4] Narod SA Rakovitch E. A comparison of the risks of in-breast recurrence after a diagnosis of DCIS or early invasive breast cancer. Curr Oncol (2014) 21(3):119. 10.3747/co.21.1892 24940092 PMC4059796

[5] Leonard GD Swain SM. Ductal carcinoma in situ, complexities and challenges. J Natl Cancer Inst (2004) 96(12):906–20. 10.1093/jnci/djh164 15199110

[6] Wiechmann L Kuerer HM. The molecular journey from ductal carcinoma in situ to invasive breast cancer. Cancer (2008) 112(10):2130–42. 10.1002/cncr.23430 18383519

[7] Ignatiadis M Sotiriou C Luminal breast cancer: from biology to treatment. Nat Rev Clin Oncol (2013) 10(9):494–506 10.1038/nrclinonc.2013.124 23881035

[8] Dawson SJ Rueda OM Aparicio S Caldas C. A new genome-driven integrated classification of breast cancer and its implications. EMBO J (2013) 32(5):617–28. 10.1038/emboj.2013.19 23395906 PMC3590990

[9] Ades F Zardavas D Bozovic-Spasojevic I Pugliano L Fumagalli D de Azambuja E Luminal B breast cancer: molecular characterization, clinical management, and future perspectives. J Clin Oncol (2014) 32(25):2794–803. 10.1200/JCO.2013.54.1870 25049332

[10] Baselga J. Critical update and emerging trends in epidermal growth factor receptor targeting in cancer. J Clin Oncol (2005) 23(11):2445–59. 10.1200/JCO.2005.11.890 15753456

[11] Zhang H Berezov A Wang Q Zhang G Drebin J Murali R ErbB receptors: from oncogenes to targeted cancer therapies. Greene J. Clin. Invest (2007) 117:2051–8 10.1172/JCI32278 PMC1934579 17671639

[12] Lehmann BD Bauer JA Chen X Sanders ME Chakravarthy AB Shyr Y Identification of human triple-negative breast cancer subtypes and preclinical models for selection of targeted therapies. J Clin Invest (2011) 121(7):2750–67. 10.1172/JCI45014 21633166 PMC3127435

[13] Allred DC. Ductal carcinoma in situ: terminology, classification, and natural history. J Natl Cancer Inst Monogr (2010) 2010(41):134–8. 10.1093/jncimonographs/lgq035 20956817 PMC5161057

[14] Morrow M Strom EA Bassett LW Dershaw DD Fowble B Harris JA Standard for the management of ductal carcinoma in situ of the breast (DCIS). CA Cancer J Clin (2002) 52:256–76. 10.3322/canjclin.52.5.256 12363325

[15] Burstein H Polyak K Wong J Susan C Kaelin CM Ductal carcinoma in situ of the breast. N Engl J Med (2004) 350:1430–41 10.1056/NEJMra031301 15070793

[16] U.S. National Library of Medicine. Genetics Home Reference: Your Guide to Understanding Genetic Conditions. (2011). Available from: http://ghr.nlm.nih.gov/gene/ERBB2http://ghr.nlm.nih.gov/gene/ERBB2

[17] Yarden Y Sliwkowski MX. Untangling the ErbB signalling network. Nat Rev Mol Cell Biol (2001) 2:127–37. 10.1038/35052073 11252954

[18] Bublil EM Yarden Y. The EGF receptor family: spearheading a merger of signaling and therapeutics. Curr Opin Cell Biol (2007) 19:124–34. 10.1016/j.ceb.2007.02.008 17314037

[19] Harris R EGF receptor ligands. Exp Cell Res (2003) 284(1):2–13 10.1016/S0014-4827(02)00105-2 12648462

[20] Garrett TP McKern NM Lou M Elleman TC Adams TE Lovrecz GO The crystal structure of a truncated ErbB2 ectodomain reveals an active conformation, poised to interact with other ErbB receptors. Mol Cell (2003) 11:495–505. 10.1016/S1097-2765(03)00048-0 12620236

[21] Cho H-S Mason K Ramyar K. Structure of the extracellular region of HER2 alone and in complex with the Herceptin Fab. Nature (2003) 421(6924):753–6. 10.1038/nature01392 12610629

[22] Nahta R. Insulin-like growth factor-I receptor/human epidermal growth factor receptor 2 heterodimerization contributes to trastuzumab resistance of breast cancer cells. Cancer Res (2005) 65(23):11118–28. 10.1158/0008-5472.CAN-04-3841 16322262

[23] Jones JT Akita R Sliwkowski M. Binding specificities and affinities of EGF domains for ErbB receptors. FEBS Lett (1999) 447:227–31. 10.1016/S0014-5793(99)00283-5 10214951

[24] Tzahar E. Eldad, bivalence of EGF-like ligands drives the ErbBsignaling network. EMBO J (1997) 16(16):4938–50. 10.1093/emboj/16.16.4938 9305636 PMC1170129

[25] Graus-Porta D Beerli RR Daly JM Hynes NE. ErbB-2, the preferred heterodimerization partner of all ErbB receptors, is a mediator of lateral signaling. EMBO J (1997) 16(7):1647–55. 10.1093/emboj/16.7.1647 9130710 PMC1169769

[26] Rimawi MF Shetty PB Weiss HL Schiff R Osborne CK Chamness GC Epidermal growth factor receptor expression in breast cancer association with biologic phenotype and clinical outcomes. Cancer (2010) 116(5):1234–42. 10.1002/cncr.24816 20082448 PMC2829330

[27] Shigematsu H Gazdar AF Somatic mutations of epidermal growth factor receptor signaling pathway in lung cancers. Int J Cancer (2006) 118(2):257–62 10.1002/ijc.21496 16231326

[28] Gan HK Kaye AH Luwor RB. The EGFRvIII variant in glioblastoma multiforme. J Clin Neurosci (2009) 16(6):748–54. 10.1016/j.jocn.2008.12.005 19324552

[29] Bronte G Terrasi M Rizzo S Sivestris N Ficorella C Cajozzo M EGFR genomic alterations in cancer: prognostic and predictive values. Front Biosci (Elite Ed) (2011) 3:879–87. 21622099 10.2741/e296

[30] Dhomen NS Mariadason J Tebbutt N Scott AM. Therapeutic targeting of the epidermal growth factor receptor in human cancer. Crit Rev Oncog (2012) 17:31–50. 10.1615/CritRevOncog.v17.i1.40 22471663

[31] Safdari Y Khalili M Farajnia S Asgharzadeh M Yazdani Y Sadeghi M. Recent advances in head and neck squamous cell carcinoma – a review. Clin Biochem (2014). 10.1016/j.clinbiochem.2014.05.066 24912050

[32] Paez JG. EGFR mutations in lung cancer: correlation with clinical response to gefitinib therapy. Science (2004) 304(5676):1497–500. 10.1126/science.1099314 15118125

[33] Sequist L Lynch TJ. EGFR tyrosine kinase inhibitors in lung cancer: an evolving story. Annu Rev Med (2008) 59:429–42. 10.1146/annurev.med.59.090506.202405 17716025

[34] Ono M Hirata A Kometani T. Sensitivity to gefitinib (Iressa, ZD1839) in non-small cell lung cancer cell lines correlates with dependence on the epidermal growth factor (EGF) receptor/extracellular signal-regulated kinase 1/2 and EGF receptor/Akt pathway for proliferation. Mol Cancer Ther (2004) 3:465–72. 15078990

[35] Ross JS Fletcher JA The HER-2/neu oncogene in breast cancer: prognostic factor, predictive factor, and target for therapy. Stem Cells (1998) 16:413–28 10.1002/stem.160413 9831867

[36] Paik S Liu ET. HER2 as a predictor of therapeutic response in breast cancer. Breast Dis (2000) 11:91–102. 15687595 10.3233/bd-1999-11108

[37] Press MF Bernstein L Thomas PA Meisner LF Zhou JY Ma Y HER-2/neu gene amplification characterized by fluorescence in situ hybridization: poor prognosis in node-negative breast carcinomas. J Clin Oncol (1997) 15:2894–904. 9256133 10.1200/JCO.1997.15.8.2894

[38] Slamon DJ Clark GM Wong SG Levin WJ Ullrich A McGuire WL. Human breast cancer: correlation of relapse and survival with amplification of the HER-2/neu oncogene. Science (1987) 235(4785):177–82. 10.1126/science.3798106 3798106

[39] Casalini P Botta L Menard S. Role of p53 in HER2-induced proliferation or apoptosis. J Biol Chem (2001) 276(15):12449–53. 10.1074/jbc.M009732200 11278558

[40] Arteaga CL Sliwkowski MX Osborne CK Perez EA Puglisi F Gianni L. Treatment of HER2-positive breast cancer: current status and future perspectives. Nat Rev Clin Oncol (2012) 9:16–32. 10.1038/nrclinonc.2011.177 22124364

[41] Altomare DA Testa JR. Perturbations of the AKT signaling pathway in human cancer. Oncogene (2005) 24(50):7455–64. 10.1038/sj.onc.1209085 16288292

[42] Hynes NE Lane HA. ERBB receptors and cancer: the complexity of targeted inhibitors. Nat Rev Cancer (2005) 5(5):341–54. 10.1038/nrc1667 15864276

[43] Sharma SV. “Oncogenic shock”: explaining oncogene addiction through differential signal attenuation. Clin Cancer Res (2006) 12(14):4392s–5s. 10.1158/1078-0432.CCR-06-0527 16857816

[44] Bray SJ. Notch signalling: a simple pathway becomes complex. Nat Rev Mol Cell Biol (2006) 7(9):678–89. 10.1038/nrm2009 16921404

[45] Carpenter G Employment of the epidermal growth factor receptor in growth factor–independent signaling pathways. J Cell Biol (1999) 146(4):697–702 10.1083/jcb.146.4.697 10459005 PMC2156131

[46] Ayyanan A Civenni G Ciarloni L Morel C Mueller N Lefort K Increased Wnt signaling triggers oncogenic conversion of human breast epithelial cells by a Notch-dependent mechanism. Proc Natl Acad Sci U S A (2006) 103(10):3799–804. 10.1073/pnas.0600065103 16501043 PMC1450156

[47] Oswald F Liptay S Adler G Schmid RM. NF-kB2 is a putative target gene of activated Notch-1 via RBP-Jk. Mol Cell Biol (1998) 18(4):2077–88. 9528780 10.1128/mcb.18.4.2077 PMC121438

[48] Osipo C Golde TE Osborne BA Miele LA. Off the beaten pathway: the complex cross talk between Notch and NF-kappaB. Lab Invest (2008) 88(1):11–7. 10.1038/labinvest.3700700 18059366

[49] Yamaguchi N Oyama T Ito E Satoh H Azuma S Hayashi M NOTCH3 signaling pathway plays crucial roles in the proliferation of ErbB2-negative human breast cancer cells. Cancer Res (2008) 68(6):1881–8. 10.1158/0008-5472.CAN-07-1597 18339869

[50] Osipo C Patel P Rizzo P Clementz AG Hao L Golde TE ErbB-2 inhibition activates Notch-1 and sensitizes breast cancer cells to a γ-secretase inhibitor. Oncogene (2008) 27(37):5019–32. 10.1038/onc.2008.149 18469855

[51] Kopan R Ilagan MXG. The canonical Notch signaling pathway: unfolding the activation mechanism. Cell (2009) 137(2):216–33. 10.1016/j.cell.2009.03.045 19379690 PMC2827930

[52] Pasinia A Henriqueb D Wilkinsona DG. The zebrafish hairy/enhancer-of-split-related gene HER6 is segmentally expressed during the early development of hindbrain and somites. Mech Dev (2001) 100:317–21. 10.1016/S0925-4773(00)00538-4 11165489

[53] Palmeirim I Henrique D Ish-Horowicz D Pourquié O. Avian hairy gene expression identifies a molecular clock linked to vertebrate segmentation and somitogenesis. Cell (1997) 91:639–48. 10.1016/S0092-8674(00)80451-1 9393857

[54] Giudicelli F Lewis J The vertebrate segmentation clock. Curr Opin Genet Dev (2004) 14(4):407–14 10.1016/j.gde.2004.06.014 15261657

[55] Lowell S Benchoua A Heavey B Smith AG. Notch promotes neural lineage entry by pluripotent embryonic stem cells. PLoS Biol (2006) 4(5):805–18. 10.1371/journal.pbio.0040121 16594731 PMC1431581

[56] Chiba S Concise review: Notch signaling in stem cell systems. Stem Cells (2006) 24(11):2437–47 10.1634/stemcells.2005-0661 16888285

[57] Artavanis-Tsakonas S. Notch signaling: cell fate control and signal integration in development. Science (1999) 284(5415):770–6. 10.1126/science.284.5415.770 10221902

[58] Li F Ambrosini G Chu EY Plescia J Tognin S Marchisio PC Control of apoptosis and mitotic spindle checkpoint by survivin. Nature (1998) 396:580–4. 10.1038/25141 9859993

[59] O’Connor DS Grossman D Plescia J Li F Zhang H Villa A Regulation of apoptosis at cell division by p34cdc2 phosphorylation of survivin. Proc Natl Acad Sci U S A (2000) 97(24):13103–7. 10.1073/pnas.240390697 11069302 PMC27185

[60] Wall NR O’Connor DS Plescia J. Janet plescia suppression of survivin phosphorylation on thr34 by flavopiridol enhances tumor cell apoptosis. Cancer Res (2003) 63:230–5. 12517802

[61] Siddiqa A Long LM Li L Marciniak RA Kazhdan I. Expression of HER-2 in MCF-7 breast cancer cells modulates anti-apoptotic proteins survivin and Bcl-2 via the extracellular signal-related kinase (ERK) and phosphoinositide-3 kinase (PI3K) signalling pathways. BMC Cancer (2008) 8(1):129. 10.1186/1471-2407-8-129 18454859 PMC2386479

[62] Lee CW Simin K Liu Q Plescia J Guha M Khan A A functional Notch-survivin gene signature in basal breast cancer. Breast Cancer Res (2008) 10(6):R97. 10.1186/bcr2200 19025652 PMC2656893

[63] Nickoloff BJ Osborne BA Miele L. Notch signaling as a therapeutic target in cancer: a new approach to the development of cell fate modifying agents. Oncogene (2003) 22(42):6598–608. 10.1038/sj.onc.1206758 14528285

[64] Reedijk M. High-level coexpression of JAG1 and Notch1 is observed in human breast cancer and is associated with poor overall survival. Cancer Res (2005) 65(18):8530–7. 10.1158/0008-5472.CAN-05-1069 16166334

[65] Ellisen L Bird J West DC. TAN-1, the human homolog of the *Drosophila* notch gene, is broken by chromosomal translocations in T lymphoblastic neoplasms. Cell (1991) 66(4):649–61. 10.1016/0092-8674(91)90111-B 1831692

[66] Fabbri G Rasi S Rossi D Trifonov V Khiabanian H Ma J Analysis of the chronic lymphocytic leukemia coding genome: role of NOTCH1 mutational activation. J Exp Med (2011) 208(7):1389–401. 10.1084/jem.20110921 21670202 PMC3135373

[67] Zagouras P Stifani S Blaumueller CM Carcangiu ML Artavanis-Tsakonas S. Alterations in Notch signaling in neoplastic lesions of the human cervix. Proc Natl Acad Sci U S A (1995) 92:6414–8. 10.1073/pnas.92.14.6414 7604005 PMC41528

[68] Akiyoshi T Nakamura M Yanai K Nagai S Wada J Koga K y-Secretase inhibitors enhance taxane-induced mitotic arrest and apoptosis in colon cancer cells. Gastroenterology (2008) 134(1):131–44. 10.1053/j.gastro.2007.10.008 18166351

[69] May R Riehl TE Hunt C Sureban SM Anant S Houchen CW. Identification of a novel putative gastrointestinal stem cell and adenoma stem cell marker, doublecortin and CaM kinase-like-1, following radiation injury and in adenomatous polyposis coli/multiple intestinal neoplasia mice. Stem Cells (2008) 26(3):630–7. 10.1634/stemcells.2007-0621 18055444

[70] Konishi J Kawaguchi KS Vo H Haruki N Gonzalez A Carbone DP y-Secretase inhibitor prevents Notch3 activation and reduces proliferation in human lung cancers. Cancer Res (2007) 67(17):8051–7. 17804716 10.1158/0008-5472.CAN-07-1022

[71] Rose SL Kunnimalaiyaan M Drenzek J Seiler N. Notch 1 signaling is active in ovarian cancer. Gynecol Oncol (2010) 117(1):130–3. 10.1016/j.ygyno.2009.12.003 20060575

[72] Nakayama K Nakayama N Jinawath N Salani R Kurman RJ Shih IeM Amplicon profiles in ovarian serous carcinomas. Int J Cancer (2007) 120(12):2613–7. 10.1002/ijc.22609 17351921

[73] Shimizu K Chiba S Saito T Kumano K Hamada Y Hirai H. Functional diversity among Notch1, Notch2, and Notch3 receptors. Biochem Biophys Res Commun (2002) 291(4):775–9. 10.1006/bbrc.2002.6528 11866432

[74] Parr C Watkins G Jiang WG. The possible correlation of Notch-1 and Notch-2 with clinical outcome and tumour clinicopathological parameters in human breast cancer. Int J Mol Med (2004) 14:779–86. 15492845 10.3892/ijmm.14.5.779

[75] O’Neill CF Urs S Cinelli C Lincoln A Nadeau RJ León R Notch2 signaling induces apoptosis and inhibits human MDA-MB-231 xenograft growth. Am J Pathol (2007) 171(3):1023–36. 10.2353/ajpath.2007.061029 17675579 PMC1959488

[76] Dotto GP. Notch tumor suppressor function. Oncogene (2008) 27(38):5115–23. 10.1038/onc.2008.225 18758480 PMC2747622

[77] Klinakis A Lobry C Abdel-Wahab O Oh P Haeno H Buonamici S A novel tumour-suppressor function for the Notch pathway in myeloid leukaemia. Nature (2011) 473(7346):230–3. 10.1038/nature09999 21562564 PMC3093658

[78] Clarke EE Churcher I Ellis S Wrigley JD Lewis HD Harrison T Intra- or intercomplex binding to the -secretase enzyme: a model to differentiate inhibitor classes. J Biol Chem (2006) 281(42):31279–89. 10.1074/jbc.M605051200 16899457

[79] Olsauskas-Kuprys R Zlobin A Osipo C. Gamma secretase inhibitors of Notch signaling. Onco Targets Ther (2013) 6:943–55. 10.2147/OTT.S33766 23901284 PMC3726525

[80] Zhang CC Pavlicek A Zhang Q Lira ME Painter CL Yan Z Biomarker and pharmacologic evaluation of the -secretase inhibitor PF-03084014 in breast cancer models. Clin Cancer Res (2012) 18(18):5008–19. 10.1158/1078-0432.CCR-12-1379 22806875

[81] Kumar JP Moses K. EGF receptor and notch signaling act upstream of eyeless/Pax6 to control eye specification. Cell (2001) 104:687–97. 10.1016/S0092-8674(01)00265-3 11257223

[82] Tsuda L Nagaraj R Zipursky SL Banerjee U. An EGFR/Ebi/Sno pathway promotes delta expression by inactivating Su(H)/SMRTER repression during inductive notch signaling. Cell (2002) 110:625–37. 10.1016/S0092-8674(02)00875-9 12230979

[83] Price JV Savenye ED Lum D Breitkreutz A. Dominant enhancers of EGR in *Drosophila melanogaster*: genetic links between the Notch and EGFR signaling pathways. Genetics (1997) 147:1139–53. 9383058 10.1093/genetics/147.3.1139 PMC1208239

[84] Kolev V Mandinova A Guinea-Viniegra J Hu B Lefort K Lambertini C EGFR signalling as a negative regulator of Notch1 gene transcription and function in proliferating keratinocytes and cancer. Nat Cell Biol (2008) 10(8):902–11. 10.1038/ncb1750 18604200 PMC2747621

[85] Purow BW Sundaresan TK Burdick MJ Kefas BA Comeau LD Hawkinson MP Notch-1 regulates transcription of the epidermal growth factor receptor through p53. Carcinogenesis (2008) 29(5):918–25. 10.1093/carcin/bgn079 18359760 PMC2902388

[86] Xie M He CS Wei SH Zhang L. Notch-1 contributes to epidermal growth factor receptor tyrosine kinase inhibitor acquired resistance in non-small cell lung cancer in vitro and in vivo. Eur J Cancer (2013) 49(16):3559–72. 10.1016/j.ejca.2013.07.007 23916913

[87] Kang JH Lee EH Park SW Chung IY MUC5AC expression through bidirectional communication of notch and epidermal growth factor receptor pathways. J Immunol (2011) 187(1):222–9 10.4049/jimmunol.1003606 21622856

[88] Esteva FJ Guo H Zhang S Santa-Maria C Stone S Lanchbury JS PTEN, PIK3CA, p-AKT, and p-p70S6K status association with trastuzumab response and survival in patients with HER2-positive metastatic breast cancer. Am J Pathol (2010) 177:1647–56 10.2353/ajpath.2010.090885 20813970 PMC2947262

[89] Gallardo A Lerma E Escuin D Tibau A Muñoz J Ojeda B Increased signalling of EGFR and IGF1R, and deregulation of PTEN/PI3K/Akt pathway are related with trastuzumab resistance in HER2 breast carcinomas. Br J Cancer (2012) 106:1367–73. 10.1038/bjc.2012.85 22454081 PMC3326683

[90] Palomero T Sulis ML Cortina M Real PJ Barnes K Ciofani M Mutational loss of PTEN induces resistance to NOTCH1 inhibition in T-cell leukemia. Nat Med (2007) 13(10):1203–10. 10.1038/nm1636 17873882 PMC2600418

[91] Veeraraghavalu K Subbaiah VK Srivastava S Chakrabarti O Syal R Krishna S. Complementation of human papillomavirus type 16 E6 and E7 by Jagged1-specific Notch1-phosphatidylinositol 3-kinase signaling involves pleiotropic oncogenic functions independent of CBF1;Su(H);Lag-1 activation. J Virol (2005) 79(12):7889–98. 10.1128/JVI.79.12.7889-7898.2005 15919944 PMC1143639

[92] Lu CH Wyszomierski SL Tseng LM Sun MH Lan KH Neal CL Preclinical testing of clinically applicable strategies for overcoming trastuzumab resistance caused by PTEN deficiency. Clin Cancer Res (2007) 13(19):5883–8. 10.1158/1078-0432.CCR-06-2837 17908983

[93] Eichhorn PJ Gili M Scaltriti M Serra V Guzman M Nijkamp W Phosphatidylinositol 3-kinase hyperactivation results in lapatinib resistance that is reversed by the mTOR/phosphatidylinositol 3-kinase inhibitor NVP-BEZ235. Cancer Res (2008) 68(22):9221–30. 10.1158/0008-5472.CAN-08-1740 19010894 PMC2587064

[94] Dong Y Li A Wang J Weber JD Michel LS. Synthetic lethality through combined Notch-epidermal growth factor receptor pathway inhibition in basal-like breast cancer. Cancer Res (2010) 70(13):5465–74. 10.1158/0008-5472.CAN-10-0173 20570903

[95] Meurette O Stylianou S Rock R Collu GM Gilmore AP Brennan K. Notch activation induces Akt signaling via an autocrine loop to prevent apoptosis in breast epithelial cells. Cancer Res (2009) 69(12):5015–22. 10.1158/0008-5472.CAN-08-3478 19491273

[96] Mungamuri SK. Survival signaling by Notch1: mammalian target of rapamycin (mTOR)-dependent inhibition of p53. Cancer Res (2006) 66(9):4715–24. 10.1158/0008-5472.CAN-05-3830 16651424

[97] Sergina NV Rausch M Wang D Blair J Hann B Shokat KM Escape from HER-family tyrosine kinase inhibitor therapy by the kinase-inactive HER3. Nature (2007) 445(7126):437–41. 10.1038/nature05474 17206155 PMC3025857

[98] Bianco R Shin I Ritter CA Yakes FM Basso A Rosen N Loss of PTEN/MMAC1/TEP in EGF receptor-expressing tumor cells counteracts the antitumor action of EGFR tyrosine kinase inhibitors. Oncogene (2003) 22(18):2812–22. 10.1038/sj.onc.1206388 12743604

[99] Engelman JA Zejnullahu K Mitsudomi T Song Y Hyland C Park JO MET amplification leads to gefitinib resistance in lung cancer by activating ERBB3, signaling. Science (2007) 316(5827):1039–43. 10.1126/science.1141478 17463250

[100] Krol J Francis RE Albergaria A Sunters A Polychronis A Coombes RC The transcription factor FOXO3a is a crucial cellular target of gefitinib (Iressa) in breast cancer cells. Mol Cancer Ther (2007) 6(12):3169–79. 10.1158/1535-7163.MCT-07-0507 18089711

[101] Han J Ma I Hendzel MJ Allalunis-Turner J. The cytotoxicity of γ-secretase inhibitor I to breast cancer cells is mediated by proteasome inhibition, not by γ-secretase inhibition. Breast Cancer Res (2009) 11(4):R57. 10.1186/bcr2347 19660128 PMC2750119

[102] Ercan D Xu C Yanagita M Monast CS Pratilas CA Montero J Reactivation of ERK signaling causes resistance to EGFR kinase inhibitors. Cancer Discov (2012) 2(10):934–47. 10.1158/2159-8290.CD-12-0103 22961667 PMC3477553

[103] Hasson P Paroush Z Crosstalk between the EGFR and other signalling pathways at the level of the global transcriptional corepressor Groucho/TLE. Br J Cancer (2006) 94(6):771–5 10.1038/sj.bjc.6603019 16508633 PMC2361374

[104] Pandya K Meeke K Clementz AG Rogowski A Roberts J Miele L Targeting both Notch and ErbB-2 signalling pathways is required for prevention of ErbB-2-positive breast tumour recurrence. Br J Cancer (2011) 105(6):796–806. 10.1038/bjc.2011.321 21847123 PMC3171020

[105] Ju JH Yang W Oh S Nam K Lee KM Noh DY HER2 stabilizes survivin while concomitantly down-regulating survivin gene transcription by suppressing Notch cleavage. Biochem J (2013) 451(1):123–34. 10.1042/BJ20121716 23323858

[106] Chan S Weng A. Notch signals positively regulate activity of the mTOR pathway in T-cell acute lymphoblastic leukemia. Blood (2007) 110:278–86. 10.1182/blood-2006-08-039883 17363738 PMC1896117

[107] Calzavara E Chiaramonte R Cesana D. Reciprocal regulation of Notch and PI3K/Akt signalling in T-ALL cells in vitro. J Cell Biochem (2008) 103:1405–12. 10.1002/jcb.21527 17849443

[108] Pradeep CR Köstler WJ Lauriola M Granit RZ Zhang F Jacob-Hirsch J Modeling ductal carcinoma in situ: a HER2–Notch3 collaboration enables luminal filling. Oncogene (2011) 31(7):907–17. 10.1038/onc.2011.279 21743488 PMC3193899

[109] Farnie G Willan PM Clarke RB Bundred NJ. Combined inhibition of ErbB1/2 and Notch receptors effectively targets breast ductal carcinoma in situ (DCIS) stem/progenitor cell activity regardless of ErbB2 status. PLoS One (2013) 8(2):e56840. 10.1371/journal.pone.0056840 23457626 PMC3572946

[110] Díaz B Yuen A Iizuka S Higashiyama S Courtneidge SA. Notch increases the shedding of HB-EGF by ADAM12 to potentiate invadopodia formation in hypoxia. J Cell Biol (2013) 201(2):279–92. 10.1083/jcb.201209151 23589494 PMC3628517

[111] Harrison H Simões BM Rogerson L Howell SJ Landberg G Clarke RB. Oestrogen increases the activity of oestrogen receptor negative breast cancer stem cells through paracrine EGFR and Notch signalling. Breast Cancer Res (2013) 15(2):R21. 10.1186/bcr3396 23497505 PMC3672803

[112] Sooman L Ekman S Andersson C Kultima HG Isaksson A Johansson F Synergistic interactions between camptothecin and EGFR or RAC1 inhibitors and between imatinib and Notch signaling or RAC1 inhibitors in glioblastoma cell lines. Cancer Chemother Pharmacol (2013) 72(2):329–40. 10.1007/s00280-013-2197-7 23736154

[113] Ma JM Meng Y Kwiatkowski D. Mammalian target of rapamycin regulates murine and human cell differentiation through STAT3/p63/Jagged/Notch cascade. J Clin Invest (2010) 120:103–14. 10.1172/JCI37964 20038814 PMC2798675

[114] Izrailita J Bermana H Dattid A. High throughput kinase inhibitor screens reveal TRB3 and MAPK-ERK/TGFβ pathways as fundamental Notch regulators in breast cancer. Proc Natl Acad Sci U S A (2013) 110:1714–9. 10.1073/pnas.1214014110 23319603 PMC3562766

[115] Sethi N Dai X Winter C. Tumor-derived Jagged1 promotes osteolytic bone metastasis of breast cancer by engaging Notch signaling in bone cells. Cancer Cell (2011) 19:192–205. 10.1016/j.ccr.2010.12.022 21295524 PMC3040415

[116] Zhang L Guo S Gonzalez-Perez RR. Notch, IL-1 and leptin crosstalk outcome (NILCO) is critical for leptin-induced proliferation, migration and VEGF/VEGFR-2 expression in breast cancer. PLoS One (2011) 6(6):e21467. 10.1371/journal.pone.0021467 21731759 PMC3121792

[117] Efferson CL Winkelmann CT Ware C Sullivan T Giampaoli S Tammam J Downregulation of Notch pathway by a -secretase inhibitor attenuates AKT/mammalian target of rapamycin signaling and glucose uptake in an ERBB2 transgenic breast cancer model. Cancer Res (2010) 70(6):2476–84. 10.1158/0008-5472.CAN-09-3114 20197467

[118] Hicks C Johnston SH diSibio G Collazo A Vogt TF Weinmaster G. Fringe differentially modulates Jagged1 and Delta1 signalling through Notch1 and Notch2. Nat Cell Biol (2000) 2:515–20. 10934472 10.1038/35019553

[119] Huang YZ Won S Ali DW Wang Q Tanowitz M Du QS Regulation of neuregulin signaling by PSD-95 interacting with ErbB4 at CNS synapses. Neuron (2000) 26:443–55. 10.1016/S0896-6273(00)81176-9 10839362

